# Supplementary material for: Agent-based model using GPS analysis for infection spread and inhibition mechanism of SARS-CoV-2 in Tokyo
Source: Sci Rep. 2022 Dec 3;12:20896. doi: 10.1038/s41598-022-25480-z (PMC9719469; doi:10.1038/s41598-022-25480-z)
Supplement: Supplementary file 1 — Supplementary Information. [file 41598_2022_25480_MOESM1_ESM.pdf]

Supplementary Information

"Agent-based model using GPS analysis for infection spread and inhibition mechanism of SARS-CoV-2 inTokyo"

Taishu Murakami[1], Shunsuke Sakuragi[1*], Hiroshi Deguchi[2], and Masaru Nakata[1]

[1]MRI Research Associates, Inc., 2-10-3 Nagata-cho, Chiyoda-ku, Tokyo 100-0014 Japan.

[2]Faculty of Commerce and Economics, Chiba University of Commerce, 1-3-1 Konodai, Ichikawa-shi, Chiba 272-8512 Japan.

*shunsuke_sakuragi@mri-ra.co.jp

Agent attribute

Table S1 shows the agent attribute. The distribution of agents is consistent with the result of national census (Ref. 15). All mesh contains the same attribute of “occupation” as facility, and number of facilities is consistent with national census (Ref. 15). In addition, agents have internal states (Table S2) which are changed depending on GPS data and result of agent-based simulation.

Table S1. Agent attribute

Agent ID	Number of agents
Age	<15
	16-64
	65<
Sex	Male
	Female
Household ID	(Same ID means living together)
Residence	Mesh ID
Workplace	Mesh ID
Occupation	Mining and quarrying of stone and gravel
	Manufacturing
	Electricity, Gas, Heat supply and Water
	Information and communications
	Transport and postal activities
	Wholesale and Retail trade
	Finance and Insurance
	Real estate and goods rental and leasing
	Scienteific research, professional and technical services
	Accomodations, eating and drinking services
	Living-related and personal services and amusement services
	Education, learning support
	Medical, health care and welfare
	Compound services
	Services, N.E.C
	Student
	None

Table S2. Internal states of agent

Infection	S
	E
	I (asymptomatic or not)
	R
Action	at home
	at office
	other (depending on institution)

Pseudocode of simulation

Before infection spread simulation, we generate agents with action information based on GPS data. The method of generate as following:

1. The values of national censuses were set as weighted random numbers to generate agents with the attributes in Table. S1.
2. The hourly action of the agents is divided into two groups, commuting agents and other agents, and set based on GPS data:
   I. Agents that commute to work or school
      i. Agents who go to work or school are selected based on the attendance probabilities by occupation obtained from the census information.
      ii. We assume that all agents at home in 3 A.M. After 3 A.M., if GPS data that moves from the home mesh to the workplace mesh at a certain time exists, the above agent is set to that GPS data and moved.
      iii. If GPS data moving from workplace mesh exists, the agent at workplace is randomly set to that GPS data and moved. The destination facilities of this agent in that mesh are determined by using weighted random number that depends on numbers of each facility group (Table S1 "Occupation") that exists in that mesh based on national census data. gents are assumed to start returning home after working for the working hours obtained from the national census data.
      iv. Note that, according to the national census data, the average commuting time in Tokyo is less than one hour, i.e., time steps of present calculation. Thus, it is not necessary to consider of commute route of agents in present calculation.
   II. Other agents
      i. By excluding GPS data presumed to be for commuting to work or school as described above, we extracted agents who do not commute to work or school.
      ii. For those agents, if GPS data to move from the home mesh exists, the agent is moved to the destination mesh based on GPS data. The destination facility in this mesh was randomly set using a weighted random number same as I.-iii.
      iii. After that, agents shopping in the around their home are set based on time-dependent probabilities obtained from the national census data, and the agents are set to go out within the same mesh.

The behavioral patterns of the agents generated by this method are in agreement with the macroscopic statistical data of the employment rate and the out-of-home rate for each time and city, and it is confirmed that the agents adopt appropriate behavioral patterns.

The infection spread simulation are performed using above agent information as following (Python like pseudocode):

```
"Read attributes of all agents"
"Set initial infectees"
for date in range(0,30,1):
    "Reading all agents' actions on 'date' from agent data"
    for time in range (0,24,1):
        "Reading the position of an agent at 'time' from agent data"
        "Initialization for facility information"
        for agent in agennt_list:
            "Counting up the number of agents per facility and the number of infected"
        for agent in agennt_list:
            "Performing infection calculation for each agent"
            "Performing infection calculation for each agent"
        "Output logs of the number of agents and infected patients per facility"
```

The minimum raw test code of main functions as following (Julia code):

```
using ArgParse
using CSV
using DataFrames
using DataStructures
using Dates
using Distributions
using JSON
using PoissonRandom
using StatsBase
using Statistics
using Society #module to use agent data

function get_args()
    settings = ArgParseSettings()
    @add_arg_table! settings begin
        "--configure", "-c"
        help = "json file."
        arg_type = String
        default = joinpath(@__DIR__, "config.json")
        "--default-input-prefix"
        help = "input directory."
```

```
        arg_type = String
        default = joinpath(@__DIR__, "../input")
        "--default-output-prefix"
        help = "output directory."
        arg_type = String
        default = joinpath(@__DIR__, "../output")
    end
    return parse_args(settings)
end

function get_config()
    args = get_args()
    config = SortedDict()
    open(args["configure"], "r") do f
        # global config
        config = JSON.parse(f; dicttype=SortedDict)
    end
    for key in ["input-prefix", "output-prefix"]
        if isa(config[key], Nothing)
            config[key] = args["default-$key"]
        end
        if ! isdir(config[key])
            throw(ErrorException(
                "$(config[key]) dose not exist."
            ))
        end
    end
    return config
end

function get_csvdata(config)
    csvdata = Dict()
    for (key, fname) in config["base"]
        csvdata[key] = CSV.read(
            joinpath(config["input-prefix"], fname),
            DataFrame,
            header=1
        )
```

```
    end
    for week = 1:config["weeks"]
        for key in ["weekday", "weekend"]
            for subkey in ["action", "time", "z"]
                fname = config["week-plan"]["$week"][key][subkey]
                if ! (fname in keys(csvdata))
                    csvdata[fname] = CSV.read(
                        joinpath(config["input-prefix"], fname),
                        DataFrame,
                        header=1
                    )
                end
            end
        end
    end
    return csvdata
end

function main()
    config = get_config()
    csvdata = get_csvdata(config)
    num_agent = size(csvdata["class"].id)[1]
    society = SocietyType(num_agent)
    for sym in [:age, :gender, :family_id, :home, :office, :job]
        setfield!(society, sym, csvdata["class"][!, sym])
    end
    society.state = csvdata["class"][!, "state_infected"];
    society.incubation = round.(Int, rand(Gamma(2.1, 2.0), num_agent))
    society.onset = rand(Poisson(7), num_agent)

    num_family = (
        findmax(csvdata["class"].family_id)[1]
        + ifelse(findmin(csvdata["class"].family_id)[1] == 0, 1, 0)
    )

    num_mesh = 5728
    mesh_range = get_range(num_mesh, comm)
    agent_range = get_range(num_agent, comm)
```

```
length_agent_range = length(agent_range)


outputname = replace("$(now())", ":"=>"-")
outputpath = joinpath(
    config["output-prefix"],
    "$(outputname)_config.json"
)
open(outputpath, "w") do f
    write(f, json(config, 4))
end


for day in 1:config["days"]
    key = ifelse((day-1) % 7 < 5, "weekday", "weekend")
    week = div(day, 7) + 1
    peak = config["week-plan"]["$week"][key]["peak"]
    action_csv =csvdata[config["week-plan"]["$week"][key]["action"]]
    time_csv = csvdata[config["week-plan"]["$week"][key]["time"]]
    z_csv = csvdata[config["week-plan"]["$week"][key]["z"]]


        outputpath1 = joinpath(
            config["output-prefix"],
            "$(outputname)_agent_state_list_day$day.csv"
        )
        open(outputpath1, "w") do f
            write(f, """time,$(join(["id_$i" for i = 0:(num_agent-1)],","))¥n""")
        end
        outputpath2 = joinpath(
            config["output-prefix"],
            "$(outputname)_num_state_list_day$day.csv"
        )
        open(outputpath2, "w") do f
            write(f, """time,S,E,I,R,Ih,Rh,Itot,Rtot¥n""")
        end
        outputpath3 = joinpath(
            config["output-prefix"],
            "$(outputname)_population_list_day$day.csv"
        )
        open(outputpath3, "w") do f
```

```
        write(f, """time,$(join([config["flag"]["place"][i] for i = 0:14],","))¥n""")
    end
    outputpath4 = joinpath(
        config["output-prefix"],
        "$(outputname)_I_Ih_list_day$day.csv"
    )
    open(outputpath4, "w") do f
        write(f, """time,$(join([config["flag"]["place"][i] for i = 0:14],","))¥n""")
    end
    outputpath5 = joinpath(
        config["output-prefix"],
        "$(outputname)_probability_list_day$day.csv"
    )
    open(outputpath5, "w") do f
        write(f, """time,$(join([config["flag"]["place"][i] for i = 0:14],","))¥n""")
    end
    outputpath6 = joinpath(
        config["output-prefix"],
        "$(outputname)_newly_infected_list_day$day.csv"
    )
    open(outputpath6, "w") do f
        write(f, """time,$(join([config["flag"]["place"][i] for i = 0:14],","))¥n""")
    end


    for t in 1:24
    if myrank == 0
        println(
            """¥rProgress: [day: $day/$(config["days"]), time: $t/24]"""
        )
    end
    rate::Array{Float32,1} = ones(num_agent)
    dict_num_infected = Dict()
    dict_num_newly_infected = Dict()
    dict_num_tot = Dict()
                dict_num_infected[0] = zeros(num_family)
    dict_num_infected[14] = zeros(num_mesh)
    dict_num_newly_infected[0] = zeros(num_family)
    dict_num_newly_infected[14] = zeros(num_mesh)
```

```
        dict_num_tot[0] = zeros(num_family)
        dict_num_tot[14] = zeros(num_mesh)
        for i = keys(config["flag"]["fac"])
            i = parse(Int, i)
            dict_num_infected[i] = zeros(num_mesh)
            dict_num_newly_infected[i] = zeros(num_mesh)
            dict_num_tot[i] = zeros(num_mesh)
        end

        for i = agent_range
            society.fac[i] = action_csv[!, "s_$(t-1)"][i]
            if society.fac[i] == 1 && society.age[i] > 0
                society.fac[i] = society.job[i]
            end
                society.loc[i] = time_csv[!, "l_$(t-1)"][i]
                k = findfirst(csvdata["area"].mesh .== society.loc[i])
                society.place[i] = society.fac[i]

            dict_num_tot[society.place[i]][k] += 1
            if society.state[i] == 2 || society.state[i] == 4
                dict_num_infected[society.place[i]][k] += 1
            end
        end

        for i = agent_range
            in_area = true
            key = string(society.place[i])
            if society.place[i] == 0
                k = society.family_id[i]
                A = 31.93*csvdata["class"].family_size[i]
                # [source](https://www.toukei.metro.tokyo.lg.jp/kurasi/2019/ku19-29.htm)
            elseif society.place[i] > 9 && society.place[i] < 14
                k = 1
                trans_rate = trans_csv[!, config["flag"]["place"][key]][t]
                if trans_rate > 0
                    A = 0.15*dict_num_tot[society.place[i]][k]/(peak*trans_rate)
                    # [source](https://www.mintetsu.or.jp/knowledge/term/157.html)
                else
```

```
                A = 10^16
            end
        else
            if indexin(society.loc[i], csvdata["area"].mesh) == nil
                in_area = false
            else
                k = findfirst(csvdata["area"].mesh .== society.loc[i])
                A = csvdata["area"].area[k]
                if A == 0
                    A = 10^12
                end
            end
        end

        if in_area
            F = z_csv[!, config["flag"]["place"][key]][1]*dict_num_infected[society.place[i]][k]/A
        else
            F = 0
        end

        rate[i] = F^2/(F^2 + 1)
        if society.state[i] == 0 # S
            if rand() < rate[i]
                society.state[i] = 1 # to E
                dict_num_newly_infected[society.place[i]][k] += 1
                society.incubation[i] += day
            end
        elseif society.state[i] == 1 # E
            if society.incubation[i] < day
                w = ifelse(rand() < config["phi-EI"], 2, 4)
                society.state[i] = w # to I or Ih
                society.onset[i] += day
                if w == 4
                    society.onset[i] = day + config["Ih-days"]
                end
            end
        elseif society.state[i] == 2 # I
            if society.onset[i] < day
```

```
                society.state[i] = 3 # to R
            end
        elseif society.state[i] == 4 # Ih
            if society.onset[i] < day
                society.state[i] = 5 # to Rh
            end
        end
    end

    list_num = SortedDict(i => 0 for i = 0:5)
        for i = 0:5
            list_num[i] = size(society.state[society.state .==  i])[1]
        end
        Itot = list_num[2] + list_num[4]
        Rtot = list_num[3] + list_num[5]
        rate_place = Dict(parse(Int, i) => 0.0 for i = keys(config["flag"]["place"]))
        rate_place_dict = Dict(parse(Int, i) => [] for i = keys(config["flag"]["place"]))
        for i = 1:num_agent
            if society.place[i] > -1
                push!(rate_place_dict[society.place[i]], rate[i])
            end
        end
        for i = keys(config["flag"]["place"])
            i = parse(Int, i)
            if length(rate_place_dict[i]) != 0 && sum(rate_place_dict[i]) > 0
                rate_place[i] = mean(rate_place_dict[i])
            end
        end

        open(outputpath1, "a") do f
            write(f, """$t,$(join(society.state, ","))¥n""")
        end
        open(outputpath2, "a") do f
            write(f, """$t,$(join(values(list_num), ",")),$Itot,$Rtot¥n""")
        end
        open(outputpath3, "a") do f
            write(f, """time,$(join([sum(dict_num_tot[parse(Int, i)]) for i = 0:14],","))¥n""")
        end
```

```
                open(outputpath4, "a") do f
                    write(f, """$t,$(join([sum(dict_num_infected[parse(Int, i)]) for i = 0:14], ","))¥n""")
                end
                open(outputpath5, "a") do f
                    write(f, """$t,$(join([rate_place[parse(Int, i)] for i = 0:14], ","))¥n""")
                end
                open(outputpath6, "a") do f
                    write(f, """$t,$(join([sum(dict_num_newly_infected[parse(Int, i)]) for i = 0:14],
",")¥n""")
                end
        end
    end
end
```
